# Supplementary material for: Process development and scale-up optimization of the SARS-CoV-2 receptor binding domain–based vaccine candidate, RBD219-N1C1
Source: Appl Microbiol Biotechnol. 2021 May 7;105(10):4153–65. doi: 10.1007/s00253-021-11281-3 (PMC8102132; doi:10.1007/s00253-021-11281-3)
Supplement: Supplementary file 1 — (PDF 234 kb) [file 253_2021_11281_MOESM1_ESM.pdf]

Applied Microbiology and Biotechnology

Supplemental Information file

Process Development and Scale-up Optimization of the SARS-CoV-2 Receptor Binding Domain-Based Vaccine Candidate, RBD219-N1C1

Jungsoon Lee[a,c]*, Zhuyun Liu[a,c], Wen-Hsiang Chen[a,c], Junfei Wei[a,c,] Rakhi Kundu[a,c], Rakesh Adhikari[a,c], Joanne Altieri Rivera[a,c], Portia M. Gillespie[a,c], Ulrich Strych[a,c], Bin Zhan[a,c], Peter J. Hotez [a, b,c,d,e], Maria Elena Bottazzi [a, b,c,d]*

[a] *National School of Tropical Medicine, Department of Pediatrics, Baylor College of Medicine, One Baylor Plaza, BCM113 Houston, TX, 77030, USA*

[b] *National School of Tropical Medicine, Department of Molecular Virology & Microbiology, Baylor College of Medicine, One Baylor Plaza, BCM113 Houston, TX, 77030, USA*

[c] *Texas Children's Hospital Center for Vaccine Development, Baylor College of Medicine, 1102 Bates Street, Houston TX, 77030, USA*

[d] *Department of Biology, College of Arts and Sciences, Baylor University, Waco TX, USA*

[e] *James A. Baker III Institute for Public Policy, Rice University, Houston TX, USA*

*Correspondence to Maria Elena Bottazzi (Email: bottazzi@bcm.edu) Tel. 832-824-0504 Fax 832-825-0549 and Jungsoon Lee (Email: jslee@bcm.edu) Tel. 832-824-0504 Fax 832-825-0549

Supplementary Fig. S1

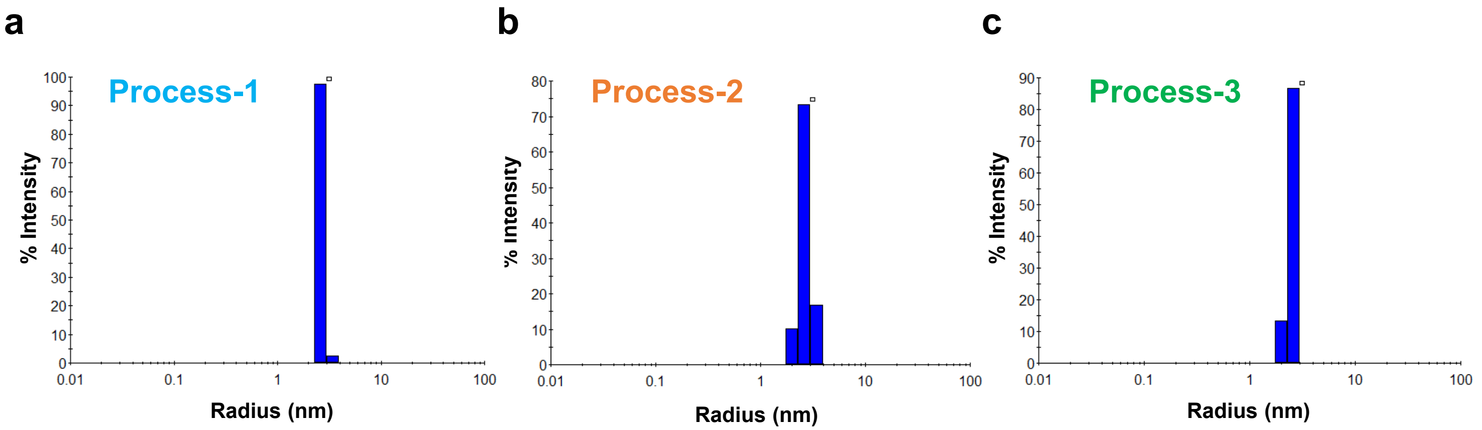

Radius (nm)	2.54±0.01
%PD	4.7±1.1
Mw (kDa)	29.75±0.43

Radius (nm)	2.59±0.01
%PD	4.3±0.8
Mw (kDa)	31±0

Radius (nm)	2.69±0.01
%PD	9.0±1.1
Mw (kDa)	34.25±0.43

Fig. S1. Dynamic light scattering results of the purified proteins from Process-1 (A), Process-2 (B), and Process-3 (C). Measured Stokes radii, polydispersity (PD), and molecular weights (Mw) are shown as an average ± SD from four independent measurements.
